# Preliminary evaluation of anti-tuberculosis potential of siderophores against drug-resistant *Mycobacterium tuberculosis* by mycobacteria growth indicator tube-drug sensitivity test

**DOI:** 10.1186/s12906-017-1665-8

**Published:** 2017-03-21

**Authors:** Karuna Gokarn, Ramprasad B. Pal

**Affiliations:** 1 Department of Microbiology, Sir Hurkisondas Nurrotumdas Medical Research Society, Mumbai, 400 002 India; 2 Caius Research Laboratory, St. Xavier’s College, Mumbai, 400 001 India

**Keywords:** Exochelin-MS, Deferoxamine-B, Xenosiderophores, Mycobacteria growth indicator tube-drug sensitivity test, Tuberculosis

## Abstract

**Background:**

Alternative treatment strategies have become essential in overcoming the problem of drug-resistant *Mycobacterium tuberculosis* (*Mtb*). In this preliminary in vitro study, the anti-tuberculosis (anti-TB) activity of exogenous iron chelators (xenosiderophores) such as Exochelin-MS (Exo-MS) and Deferoxamine-B (DFO-B) was evaluated against ten multi-drug-resistant (MDR) and seven pyrazinamide-resistant (*PZA* ^*R*^) *Mtb* isolates.

**Methods:**

Mycobacteria Growth Indicator Tube-Drug Susceptibility Test was used to assess the anti-TB effect of Exo-MS or DFO-B individually and their combinations with isoniazid (*INH*), rifampicin (*RIF*) and pyrazinamide (*PZA*).

**Results:**

For the MDR-*Mtb* isolates, Exo-MS alone inhibited two out of the five isolates tested. Whereas, DFO-B alone inhibited nine out of the ten MDR isolates tested. For *PZA*-resistant *Mtb* isolates, both Exo-MS and DFO-B individually inhibited five out of the seven isolates. The MIC of Exo-MS in combination with *INH*, *RIF* and *PZA* remained the same. The MIC of DFO-B decreased when tested in combination with *INH*, *RIF* and *PZA*.

**Conclusions:**

Exo-MS and DFO-B were shown to have activity against drug-resistant *Mtb* isolates. Therefore, these xenosiderophores may be useful adjuncts to antibiotics in overcoming the problem of drug-resistant *Mtb* in clinical setting.

## Background

Tuberculosis (TB) is one of the leading infectious diseases in the world today. *Mycobacterium tuberculosis* (*Mtb*), the causative agent of TB, is mainly transmitted from person to person through aerosols. Infections caused by TB bacteria are usually treated with the first-line antituberculosis drugs, namely, isoniazid (*INH*), rifampicin (*RIF*), ethambutol (*EMB*), pyrazinamide (*PZA*), and streptomycin (*STR*).

However, *Mtb* bacteria have acquired resistance over the years to many of these drugs due to inappropriate use, mutations in *Mtb*, etc. These factors have resulted in the emergence of multi-drug-resistant TB (MDR-TB) and extensively-drug-resistant TB (XDR-TB), which have rendered current treatment strategies ineffective.

In MDR-TB, the organisms develop resistance to *INH* and *RIF*, the two most important primary drugs. In XDR-TB, the bacteria are resistant to the first-line as well as to the second-line anti-TB drugs. XDR-TB develops in about 9% of MDR-TB patients and is more challenging to treat [1]. In 2014, globally 4,80,000 cases of MDR-TB were reported with mortality of 1,90,000. XDR-TB patients have been identified in about 100 countries. As per this WHO report, India, along with other third world countries is a high-burden country for TB. With the aim of ending TB by 2035, WHO has initiated an “End TB Strategy” [2].

Iron is a vital nutrient for all living organisms necessary for life-sustaining cellular processes such as cell growth, DNA synthesis, electron transport, oxygen transport, etc. The mammalian host maintains low levels of iron using its iron-binding proteins; about 10^−18^ M of circulating free ferric (Fe^3+^) ions. Microorganisms synthesize small molecules called siderophores (Greek “iron-carrier”) which scavenge iron from the host to promote their own growth [3]. Siderophore-Fe^3+^ ion complexes that are formed are then transported intracellularly via specific outer membrane receptor proteins by bacteria.

If Fe^3+^ ion is complexed with xenosiderophores (not “self”) for a given microorganism, iron uptake may be affected due to the absence of specific receptors in most bacteria. A decrease in the availability of iron would probably be detrimental to the growth of pathogens including the drug-resistant ones. A phytosiderophore extracted from plant root washings has been reported to inhibit the in vitro growth of H_37_ Ra strain of *Mtb* [4].

*M. smegmatis* produces three siderophores: mycobactin S (S for *smegmatis*), carboxymycobactin S and Exochelin MS (Exo-MS; MS for *M. smegmatis*). *Mtb* expresses mycobactin T (T for *tuberculosis*) and carboxymycobactin T. However, it does not produce exochelin. Therefore, Exo-MS is a xenosiderophore for *Mtb*. Similarly, the commercially available Deferoxamine mesylate (DFO-B), originally extracted from *Streptomyces pilosus*, is also a xenosiderophore for *Mtb*. Desferri forms of Exo-MS and DFO-B were tested for their anti-TB potential alone and in combination with drugs against MDR and *PZA* ^*R*^-*Mtb* isolates by the Mycobacteria Growth Indicator Tube – Drug Susceptibility Test (MGIT^TM^-DST) [5].

MGIT-DST is a qualitative test used for antimycobacterial susceptibility testing of *Mtb* developed by Becton Dickinson. A fluorescent oxygen-quenched sensor (Tris 4, 7-diphenyl-1, 10 phenanthroline ruthenium chloride pentahydrate) embedded in silicone is present at the base of a tube. The principle underlying the test is that the initial concentration of dissolved oxygen in the Middlebrook 7H9 broth quenches the emission of fluorescence from this sensor and therefore is not visualized under UV light. However, when actively growing *Mtb* bacilli consume the available oxygen, the sensor fluoresces and is visualized under UV light. The intensity of this fluorescence is directly proportional to the amount of oxygen consumed by the *Mtb* bacilli for growth. The amount of oxygen utilized by *Mtb* for growth in the medium is monitored by the BACTEC MGIT system which automatically interprets results as ‘S’(susceptible) or ‘R’ (resistant) based on the extent of fluorescence [5].

## Methods

### Extraction and purification of Exo-MS from *M. smegmatis* mc^2^155

*M. smegmatis* was grown in an iron-deficient medium [6, 7] for 10 days at 37 °C with aeration [For details of Exo-MS extraction, refer to Gokarn et al., 2017, BCAM DOI:10.1186/s12906-017-1657-8]. Briefly, Exo-MS was recovered from the culture supernatant using the benzyl alcohol extraction procedure [8]. Purification was carried out on an alumina column [9], which removed most of the hydrophobic impurities; Exo-MS was then eluted using a mixture of methanol and formic acid. HR-LCMS was carried out to determine the purity of Exo-MS.

### Determination of in vitro effect of Exo-MS and DFO-B on MDR and *PZA*-resistant *Mtb* isolates by MGIT-DST method

i. *Clinical isolates*: To identify the drug-resistant status of the clinical *Mtb* isolates, MGIT-DST was carried out using multiple drugs that include streptomycin, *INH*, *RIF* and ethambutol (SIRE) along with *PZA* and other antibiotics. The isolates resistant to *INH* and *RIF* – termed as MDR isolates – were used in this study. Besides these, isolates showing resistance to *PZA* were also used. The revival of the *Mtb* isolates was done in Middlebrook 7H9 broth supplemented with MGIT PANTA (reconstituted with MGIT growth supplement).
  Five MDR-*Mtb* isolates were used for testing the anti-TB activity of Exo-MS alone and its combination with *INH* and *RIF*. Ten MDR-*Mtb* isolates were used for testing the activity of DFO-B alone and its combination with *INH* and *RIF*. Five of the MDR-*Mtb* isolates were also resistant to *PZA*. These five and two non-MDR isolates resistant to *PZA* (making a total of seven *PZA*-resistant *Mtb* isolates) were used to evaluate the activity of Exo-MS and DFO-B individually and in combination with *PZA*.
ii. *Method*: MGIT-DST was used to determine the anti-TB activity of the siderophores and their combination with drugs. The test was conducted as per the standard protocol recommended in the BACTEC MGIT system manual [5].
iii. *Testing of siderophores and their combinations*: Exo-MS isolated in our laboratory was used for this study at a concentration of 19 mg/mL [Gokarn et al., 2017, BCAM DOI:10.1186/s12906-017-1657-8]. The concentration of Exo-MS and its combinations with *INH* and *RIF* used against MDR isolates are shown in Table 1. Similarly, its concentration alone and in combination with *PZA* against *PZA*-resistant isolates are shown in Table 2.

**Table 1:** Protocol for MGIT-DST of MDR-*Mtb* with Exo-MS alone and in combination with *INH* and *RIF*

No	Drug	Initial Concentration of the drug preparation	Volume added to MGIT for Test	Final Concentration in MGIT tube except GC	No. of Middle Brook tubes	
					GC	MDR
1	*INH*	8.3 μg/mL	100 μL	0.1 μg/mL	1	1
2	*RIF*	83 μg/mL	100 μL	1.0 μg/mL		1
3	Exo-MS + *INH*	19 mg/mL + 8.3 μg/mL	50 μL + 100 μL	0.125 mg/mL and 0.1 μg/mL		1
4	Exo-MS + *INH*	19 mg/mL + 8.3 μg/mL	100 μL + 100 μL	0.25 mg/mL and 0.1 μg/mL		1
5	Exo-MS + *INH*	19 mg/mL + 8.3 μg/mL	200 μL + 100 μL	0.5 mg/mL and 0.1 μg/mL	1	1
6	Exo-MS + *RIF*	19 mg/mL + 83 μg/mL	50 μL + 100 μL	0.125 mg/mL and 1.0 μg/mL		1
7	Exo-MS + *RIF*	19 mg/mL + 83 μg/mL	100 μL + 100 μL	0.25 mg and 1.0 μg/mL		1
8	Exo-MS + *RIF*	19 mg/mL + 83 μg/mL	200 μL + 100 μL	0.5 mg and 1.0 μg/mL		1
9	Exo-MS	19 mg/mL	50 μL	0.125 mg/mL	1	1
10	Exo-MS	19 mg/mL	100 μL	0.25 mg/mL		1
11	Exo-MS	19 mg/mL	200 μL	0.5 mg/mL		1
12	Exo-MS+ FeCl_3_	19 mg/mL + 80 mg/mL	200 μL + 50 μL	0.5 mg/mL and 0.5 mg/mL		1

**Table 2:** Protocol for MGIT-DST for *PZA* ^*R*^-*Mtb* with Exo-MS alone and in combination with *PZA*

No	Drug	Initial Concentration of the drug preparation	Volume added to MGIT for Test	Final Concentration in MGIT tube except GC	No. of *PZA* tubes	
					GC	*PZA* ^*R*^
1	*PZA*	8 mg/mL	100 μL	0.1 mg/mL	1	1
2	Exo-MS + *PZA*	19 mg/mL + 8 mg/mL	50 μL + 100 μL	0.125 mg/mL and 0.1 mg/mL		1
3	Exo-MS + *PZA*	19 mg/mL + 8 mg/mL	100 μL + 100 μL	0.25 mg/mL and 0.1 mg/mL		1
4	Exo-MS + *PZA*	19 mg/mL + 8 mg/mL	200 μL + 100 μL	0.5 mg/mL and 0.1 mg/mL		1
5	Exo-MS	19 mg/mL	50 μL	0.125 mg/mL and 0.1 mg/mL	1	1
6	Exo-MS	19 mg/mL	100 μL	0.25 mg/mL		1
7	Exo-MS	19 mg/mL	200 μL	0.5 mg/mL		1
8	Exo-MS+ FeCl_3_	19 mg/mL + 80 mg/mL	200 μL + 50 μL	0.5 mg/mL and 0.5 mg/mL		1
  A working stock of DFO-B (160 mg/mL) was prepared from 250 mg/mL stock solution before use. The concentration of DFO-B and its combinations with *INH* and *RIF* used against MDR isolates are shown in Table 3. Similarly, its concentration alone and in combination with *PZA* against *PZA*-resistant isolates are shown in Table 4.

**Table 3:** Protocol for MGIT-DST of MDR-*Mtb* with DFO-B alone and in combination with *INH* and *RIF*

No	Drug	Initial Concentration of the drug preparation	Volume added to MGIT for Test	Final Concentration in MGIT tube except GC	No. of Middle Brook tubes	
					GC	MDR
1	*INH*	8.3 μg/mL	100 μL	0.1 μg/mL	1	1
2	*RIF*	83 μg/mL	100 μL	1.0 μg/mL		1
3	DFO-B + *INH*	160 mg/mL + 8.3 μg/mL	12.5 μL + 100 μL	0.25 mg/mL and 0.1 μg/mL		1
4	DFO-B + *INH*	160 mg/mL + 8.3 μg/mL	25 μL and 100 μL	0.5 mg/mL and 0.1 μg/mL		1
5	DFO-B + *INH*	160 mg/mL + 8.3 μg/mL	50 μL and 100 μL	1.0 mg/mL and 0.1 μg/mL	1	1
6	DFO-B + *RIF*	160 mg/mL + 83 μg/mL	12.5 μL + 100 μL	0.25 mg/mL and 1.0 μg/mL		1
7	DFO-B + *RIF*	160 mg/mL + 83 μg/mL	25 μL + 100 μL	0.5 mg/mL and 1.0 μg/mL		1
8	DFO-B + *RIF*	160 mg/mL + 83 μg/mL	50 μL + 100 μL	1.0 mg/mL and 1.0 μg/mL		1
9	DFO-B	160 mg/mL	12.5 μL	0.25 mg/mL	1	1
10	DFO-B	160 mg/mL	25 μL	0.5 mg/mL		1
11	DFO-B	160 mg/mL	50 μL	1.0 mg/mL		1
12	DFO-B + FeCl_3_	160 mg/mL + 80 mg/mL	50 μL + 50 μL	1.0 mg/mL and 0.5 mg/mL		1

**Table 4:** Protocol for MGIT-DST for *PZA* ^*R*^-*Mtb* with DFO-B alone and in combination with *PZA*

No	Drug	Initial Concentration of the drug preparation	Volume added to MGIT for Test	Final Concentration in MGIT tube except GC	No. of *PZA* tubes	
					GC	*PZA* ^*R*^
1	*PZA*	8 mg/mL	100 μL	0.1 mg/mL	1	1
2	DFO-B + *PZA*	160 mg/mL + 8 mg/mL	12.5 μL + 100 μL	0.25 mg/mL and 0.1 mg/mL		1
3	DFO-B + *PZA*	160 mg/mL + 8 mg/mL	25 μL + 100 μL	0.5 mg/mL and 0.1 mg/mL		1
4	DFO-B + *PZA*	160 mg/mL + 8 mg/mL	50 μL + 100 μL	1.0 mg/mL and 0.1 mg/mL		1
5	DFO-B	160 mg/mL	12.5 μL	0.25 mg/mL	1	1
6	DFO-B	160 mg/mL	25 μL	0.5 mg/mL		1
7	DFO-B	160 mg/mL	50 μL	1.0 mg/mL		1
8	DFO-B + FeCl_3_	160 mg/mL + 80 mg/mL	50 μL + 50 μL	1.0 mg/mL and 0.5 mg/mL		1
  For each isolate, one set of tubes was supplemented with excess ferric ammonium citrate as control to ascertain whether the growth inhibition was solely due to iron deprivation.
  All the tubes were mixed by inverting several times and then placed in set carriers before transferring them into the MGIT instrument maintained at 37 °C. The first tube in the set carrier is always the Growth Control (GC) tube. In MGIT-DST, the GC should have a Growth Unit (GU) value of 400 or more. If the GU value of the drug-containing tube is <100, the interpretation “S” is read by the MGIT instrument. If the GU value of the drug-containing tube is ≥ 100, the reading “R” is given by the instrument [5]. When the GU value of the GC was 400 or greater, the tubes were removed, scanned and a report was generated.
iv *Test for bactericidal or bacteriostatic effect of the siderophores*: Determination of minimum bactericidal concentration was carried out to test whether the concentration of the siderophores used had a bacteriostatic (inhibiting), or bactericidal (killing) effect. For this test, 0.5 mL aliquots from the tubes showing GU value <100 after 14 days were re-inoculated into fresh Middlebrook 7H9 broth without any siderophores and antibiotics; the tubes were incubated in the MGIT instrument for 35 days.

## Results

### Extraction and purification of Exo-MS from *M. smegmatis* mc^2^155

HR-LC/MS analysis confirmed the presence of Exo-MS [Gokarn etal., 2017, BCAM DOI:10.1186/s12906-017-1657-8]. Since Exo-MS was used to chelate iron, it was isolated in desferri form, i.e., not saturated with iron. DFO-B was obtained in desferri form.

### Inhibitory effect of Exo-MS alone and in combination with *INH* and *RIF* on MDR-*Mtb* isolates in vitro

Table 5 shows the GU values of Exo-MS, Exo-MS + *INH*, and Exo-MS + *RIF* combinations on five different isolates of MDR-*Mtb*. For two of the five MDR-*Mtb* isolates, MIC of Exo-MS alone was 0.5 mg/mL. No concentration below this was inhibitory in combination with *INH* or *RIF*.

**Table 5:** MGIT-DST of MDR-*Mtb* with Exo-MS alone and in combination with *INH* and *RIF*

MDR- *Mtb* (*INH* ^*R*^ + *RIF* ^R^) Isolate	Exo-MS (mg/mL)			Exo-MS (mg/mL) + *INH* 0.1 μg/mL			Exo-MS (mg/mL) + *RIF* 1.0 μg/mL			Ferric citrate + Exo-MS (mg/mL)
	0.125	0.25	0.5	0.125	0.25	0.5	0.125	0.25	0.5	0.5
1	400	400	400	400	400	400	400	400	400	400
2	400	400	0	400	400	0	400	400	0	400
3	400	400	0	400	400	0	400	400	12	400
4	400	400	400	400	400	400	400	400	400	400
5	400	400	253	400	400	105	400	400	131	400
INFERENCE	R	R	S *n* = 2	R	R	S *n* = 2	R	R	S *n* = 2	R

GU value of GC for all five isolates was 400. GU value in *INH*- and *RIF*-containing medium for all isolates was also 400. *R* resistant, *S* Susceptible. If the GU value of the drug-containing tube is ≥100, the interpretation is Resistant by the MGIT instrument. If the GU value of the drug tube is <100, the interpretation is Susceptible (*n* = no. of isolates susceptible)

Inhibitory effect of Exo-MS, Exo-MS + *INH*, Exo-MS + *RIF* on five clinical isolates of MDR-*Mtb* (*INH*-, *RIF*- and *PZA*-resistant). The medium used was Middlebrook 7H9 broth, pH = 7 incubated at 37 °C for14 days in the MGIT instrument

### Inhibitory effect of DFO-B alone and in combination with *INH* and *RIF* on MDR-*Mtb* isolates in vitro

Table 6 shows the GU values of DFO-B and DFO-B + *INH*, DFO-B + *RIF* combinations against the ten different isolates of MDR-*Mtb* isolates. For four MDR isolates, MIC of DFO-B alone was 0.5 mg/mL. For five other MDR isolates, MIC of DFO-B alone was 1.0 mg/mL. For one of these five isolates, MIC of DFO-B + *INH* and DFO-B + *RIF* combinations decreased to 0.5 mg/mL.

**Table 6:** MGIT-DST of MDR-*Mtb* with DFO-B alone and in combination with *INH* and *RIF*

MDR- *Mtb* (*INH* ^*R*^ + *RIF* ^R^) Isolate	DFO-B (mg/mL)			DFO-B (mg/mL) + *INH* 0.1 μg/mL			DFO-B (mg/mL) + *RIF* 1.0 μg/mL			Ferric citrate + DFO-B (mg/mL)
	0.25	0.5	1.0	0.25	0.5	1.0	0.25	0.5	1.0	1.0
1	400	400	0	400	400	0	400	400	0	400
2	400	400	0	400	400	0	400	400	0	400
3	400	0	0	400	0	0	400	0	0	400
4	400	0	0	400	0	0	400	0	0	400
5	400	400	400	400	400	400	400	400	400	400
6	400	400	0	400	0	0	400	0	0	400
7	400	400	0	400	400	0	400	400	0	400
8	400	0	0	400	0	0	400	0	0	400
9	400	0	0	400	0	0	400	0	0	400
10	400	400	0	400	400	0	400	400	0	400
INFERENCE	R	S *n* = 4	S *n* = 9	R	S *n* = 5	S *n* = 9	R	S *n* = 5	S *n* = 9	R

GU value of GC for all ten isolates was 400. GU value in *INH*- and *RIF*-containing medium for all isolates was also 400. *R* resistant, *S* susceptible. If the GU value of the drug-containing tube is ≥100, the interpretation is Resistant by the MGIT instrument. If the GU value of the drug tube is <100, the interpretation is Susceptible (*n* = no. of isolates susceptible)

Inhibitory effect of DFO-B, DFO-B + *INH*, DFO-B + *RIF* on ten different clinical isolates of MDR-*Mtb* (*INH*-, *RIF*- and *PZA*-resistant). The medium used was Middlebrook 7H9 broth, pH = 7 incubated at 37 °C for 14 days in the MGIT instrument

So, a total of nine out of ten MDR-*Mtb* isolates were inhibited by DFO-B alone (and its combinations).

### Inhibitory effect of Exo-MS and DFO-B alone and in combination with *PZA* on *PZA*-resistant *Mtb* isolates in vitro

Table 7 shows the GU values of Exo-MS and Exo-MS + *PZA* combination on seven *PZA*-resistant isolates.

**Table 7:** MGIT-DST of *PZA* ^*R*^-*Mtb* with Exo-MS alone and in combination with *PZA*

*Mtb* Isolate	Exo-MS (mg/mL)			Exo-MS (mg/mL) + *PZA* 100 μg/mL			Ferric citrate + Exo-MS (mg/mL)
	0.125	0.25	0.5	0.125	0.25	0.5	0.5
*PZA* ^*R*^ isolate 1	0	0	0	0	0	0	400
*PZA* ^*R*^ isolate 2	0	0	0	0	0	0	400
MDR + *PZA* ^*R*^ isolate 3	400	0	0	400	0	0	400
MDR + *PZA* ^*R*^ isolate 4	400	400	400	400	400	400	400
MDR + *PZA* ^*R*^ isolate 5	400	25	0	400	0	0	400
MDR + *PZA* ^*R*^ isolate 6	400	317	0	400	237	0	400
MDR + *PZA* ^*R*^ isolate 7	400	400	400	400	386	253	400
INFERENCE	S *n* = 2	S *n* = 4	S *n* = 5	S *n* = 2	S *n* = 4	S *n* = 5	R

GU value of GC for all seven isolates was 400. GU value in *PZA*-containing medium for all isolates was also 400. *R* resistant, *S* susceptible. If the GU value of the drug-containing tube is ≥100, the interpretation is Resistant by the MGIT instrument. If the GU value of the drug tube is <100, the interpretation is Susceptible (*n* = no. of isolates susceptible)

Inhibitory effect of Exo-MS and Exo-MS + *PZA* on seven *PZA* ^*R*^ isolates: The medium used was Middlebrook 7H9 broth, pH = 5.9 incubated at 37 °C for14 days in the MGIT instrument

For the two non-MDR *PZA*-resistant isolates, MIC of Exo-MS alone was 0.125 mg/mL. For two MDR *PZA*-resistant isolates, the MIC was 0.25 mg/mL. For one MDR *PZA*-resistant isolate, the MIC was 0.5 mg/mL.

Thus, of the seven *PZA*-resistant *Mtb* isolates tested, five were inhibited by Exo-MS alone. Exo-MS + *PZA* combination did not change the MIC.

Table 8 shows the GU values of DFO-B and DFO-B + *PZA* combination on seven *PZA*-resistant isolates. For the two non-MDR *PZA*-resistant isolates, MIC of DFO-B alone was 0.5 mg/mL and it decreased to 0.25 mg/mL for DFO-B + *PZA* combination. For three of the five MDR *PZA*-resistant isolates, MIC of DFO-B alone was 1.0 mg/mL. For two of these three isolates, MIC of DFO-B + *PZA* combination decreased to 0.25 mg/mL. Thus, of the seven isolates of *PZA*-resistant *Mtb*, five were susceptible to DFO-B and DFO-B + *PZA*.

**Table 8:** MGIT-DST of *PZA* ^*R*^-*Mtb* with DFO-B alone and in combination with *PZA*

*Mtb* Isolate	DFO-B (mg/mL)			DFO-B (mg/mL) + *PZA* 100 μg/mL			Ferric citrate + DFO-B (mg/mL)
	0.25	0.5	1.0	0.25	0.5	1.0	1
*PZA* ^*R*^ isolate 1	209	41	0	0	0	0	400
*PZA* ^*R*^ isolate 2	129	0	0	0	0	0	400
MDR + *PZA* ^*R*^ isolate 3	400	400	0	7	0	0	400
MDR + *PZA* ^*R*^ isolate 4	400	400	179	400	400	400	400
MDR + *PZA* ^*R*^ isolate 5	400	400	0	0	0	0	400
MDR + *PZA* ^*R*^ isolate 6	400	400	400	400	400	400	400
MDR + *PZA* ^*R*^ isolate 7	400	400	0	400	400	0	400
INFERENCE	R	S *n* = 2	S *n* = 5	S *n* = 4	S *n* = 4	S *n* = 5	R

GU value of GC for all seven isolates was 400. GU value in *PZA*-containing medium for all isolates was also 400. *R* resistant, *S* susceptible. If the GU value of the drug-containing tube is ≥100, the interpretation is Resistant by the MGIT instrument. If the GU value of the drug tube is <100, the interpretation is Susceptible (*n* = no. of isolates susceptible)

Inhibitory effect of DFO-B and DFO-B + *PZA* on seven *PZA* ^*R*^ isolates: The medium used was Middlebrook 7H9 broth, pH = 5.9 incubated at 37 °C for 14 days in the MGIT instrument

The control tubes with ferric citrate added showed GU value of 400 for all the isolates.

### Bacteriostatic or bactericidal effect of the siderophores in vitro

When the inhibited isolates showing GU value of <100 were re-inoculated into fresh siderophore-free and drug-free Middlebrook 7H9 medium, the distinction between bacteriostatic and bactericidal effect of the siderophores (and their combinations) could be inferred based on the GU values. If the GU value of re-inoculated tube was <100, the original siderophore-drug combination was considered bactericidal. If the GU value of re-inoculated tube was 400, the original siderophore-drug combination was considered bacteriostatic.

After 14 days of re-inoculation into fresh medium, the GU values were <100 for two isolates inhibited by Exo-MS + *INH*, three of the nine isolates inhibited by DFO-B + *RIF* and three of the five isolates inhibited by DFO-B + *PZA*. However, at the end of 35 days, all concentrations of siderophores and their combinations with drugs were found to be bacteriostatic for all isolates, with GU values of 400.

## Discussion

Tubercle bacilli acquire iron from a mammalian host for their growth. Excess iron in vivo promotes *Mtb* infection and may impair macrophage function affecting innate immunity. A study using female Balb/C mouse infected with *Mtb* showed that iron overloading significantly reduced the bactericidal activity of *INH* and completely neutralized the bacteriostatic activity of ethambutol [10].

There are various strategies that the human body has adapted to safeguard the iron. Iron retention by the reticuloendothelial system [11] and mild anemia which is a common occurrence in TB patients are examples of host mechanisms to ensure iron-deficient conditions in the body [12, 13]. Though these natural processes exist, restricting the spread of MDR-*Mtb* in the human population is still a major problem.

MDR-*Mtb* strains are defined as those that are resistant to *INH* and *RIF*, both of which are important anti-TB drugs. *PZA* is also a first-line anti-TB drug. All the three drugs are bactericidal to susceptible strains of *Mtb* and hence were chosen for this study. Ethambutol was not selected since it is bacteriostatic to susceptible *Mtb* strains.

For the first time, MIC of siderophores has been determined using the MGIT-DST method for drug-resistant *Mtb* isolates. It is important to note that the anti-TB activities of Exo-MS and DFO-B by themselves were significant.

Inhibitory effect of Exo-MS on MDR-*Mtb* isolates was determined in the Middlebrook 7H9 medium with a neutral pH. Two of the five MDR-*Mtb* isolates tested were susceptible to Exo-MS. The same numbers were susceptible to its combination with *INH* and *RIF*. Due to limited availability of Exo-MS, only five isolates of MDR-*Mtb* could be tested with it.

When the inhibitory effect of DFO-B on MDR-*Mtb* isolates was determined, nine out of ten isolates of MDR-*Mtb* tested were found to be susceptible to DFO-B. The same numbers were susceptible to its combination with *INH* and *RIF*, though at a lower DFO-B concentration for some.

Inhibitory effect of Exo-MS and DFO-B on *PZA*-resistant *Mtb* isolates was determined in a modified Middlebrook 7H9 medium with pH 5.9. The standard growth medium used for testing *INH* and *RIF* is not used for *PZA*, since *PZA* requires acidic pH for activity in vitro. Five of the seven *PZA*-resistant *Mtb* isolates were susceptible to Exo-MS alone. There was no change in the MIC in combination with *PZA*. Similarly, five of these isolates were susceptible to DFO-B alone and also to DFO-B + *PZA*. For four of these isolates, the MIC of DFO-B decreased to half when used in combination with *PZA*.

A significant observation was that the inhibitory activity of the siderophores was abolished when excess iron was added in the medium. This proves that the anti-TB effect of the siderophores is solely due to their ability to deprive pathogens of iron. This makes it imperative to use the desferri form of siderophores in such studies.

For all isolates, the siderophore concentrations used were found to be bacteriostatic. This could be beneficial in restricting multiplication of the pathogen in vivo, thus participating in controlling the infection.

The tubercle bacilli down-regulate iron-containing proteins during iron-deficiency [14]. Therefore, iron-deprivation by Exo-MS and DFO-B may result in inhibition or inactivation of proteins and enzymes involved in vital functions required for drug resistance such as cell wall integrity. Perhaps Exo-MS and DFO-B may act as facilitators for antibiotics across the cell membrane due to increased cell permeability. Therefore, in the presence of exogenous iron chelators, drug-resistant *Mtb* could once again become susceptible to the same drugs.

Middlebrook 7H9 medium contains high levels of iron which may not represent true physiological concentrations of ferric ions. It could be one of the reasons why the MIC of the siderophores was high in vitro. Such susceptibility testing predicts the possible antimicrobial agent for the treatment of an infection. The clinical outcome may not be the same due to factors such as host physiology or other interventions, which cannot be simulated in laboratory tests. In vivo studies are required to validate these most promising in vitro results. While DFO-B is already approved for use to treat iron overload in thalassemic patients, safety of Exo-MS for therapeutic use needs to be determined.

We have shown in another study that Exo-MS and DFO-B do not have any cytotoxic effects in vitro on normal mammalian cell lines, such as human embryonic kidney cell line HEK-293 and mouse fibroblast cell line NIH/3 T3 [Gokarn etal., 2017, BCAM DOI:10.1186/s12906-017-1657-8]. From the results of the anti-TB activity as well as the effect on normal mammalian cells, it can be concluded that Exo-MS at 0.5 mg/mL and DFO-B at 1.0 mg/mL selectively inhibit *Mtb* without significantly harming normal mammalian cells.

## Conclusions

Our study is the first ever investigation done to evaluate siderophores as potential agents against drug-resistant *Mtb* by MGIT-DST method. Since the work was exploratory in nature, few drug-resistant isolates of *Mtb* were used. Nonetheless, it has provided a “proof of concept” that exogenous siderophores such as Exo-MS and DFO-B could be valuable additions to fight drug-resistant *M. tuberculosis*. In view of the encouraging preliminary results, new in vitro study with large number of *M. tuberculosis* isolates can be carried out.

## Abbreviations

DFO-B: Deferoxamine-B
Exo-MS: Exochelin-MS
GC: Growth control
GU: Growth unit
HR-LCMS: High resolution liquid chromatography and mass spectrometry
INH: Isoniazid
MDR: Multi-drug-resistant
MGIT-DST: Mycobacteria growth indicator tube-drug sensitivity test
MIC: Minimum inhibitory concentration
Mtb: Mycobacterium tuberculosis
PZA: Pyrazinamide
R: Resistant
RIF: Rifampicin
S: Susceptible
TB: Tuberculosis
WHO: World health organization
XDR: Extensively-drug-resistant
